# Knowledge, attitudes, and practices toward colorectal cancer lifestyle risk factors among adults in Saudi Arabia

**DOI:** 10.3389/fnut.2025.1507563

**Published:** 2025-04-08

**Authors:** Areej Ali Alkhaldy

**Affiliations:** Department of Clinical Nutrition, Faculty of Applied Medical Sciences, King Abdulaziz University, Jeddah, Saudi Arabia

**Keywords:** nutrition, knowledge, attitudes, practices, colorectal cancer

## Abstract

**Background:**

Despite an apparent increase in early-onset colorectal cancer (CRC) in Saudi Arabia, with the majority of patients being diagnosed at an advanced disease stage, no previous assessment of the knowledge, attitudes, and practices (KAP) toward its dietary and lifestyle-related risk factors has been reported. The aim of this study was to investigate the KAP levels with respect to these risk factors for CRC and examine possible associations between the studied variables among the Saudi population.

**Methods:**

This cross-sectional study involved 1,040 participants aged 18 years or older. Data were collected by convenience sampling via a self-administered online questionnaire in Saudi Arabia between June and December 2023.

**Results:**

A majority of participants (77.8%) displayed low knowledge about the dietary and lifestyle-related risk factors for CRC, while only 22.2% possessed high knowledge. Similarly, 78.6% of participants exhibited negative attitudes toward these risk factors, with just 21.4% having positive attitudes. Furthermore, 75.0% of participants reported engaging in poor practices, leaving only 25.0% demonstrating good practices related to CRC risk factors.

**Conclusions:**

The findings of this study indicate insufficient KAP levels toward dietary and lifestyle-related risk factors for CRC in Saudi Arabia, highlighting the urgent need for nationwide initiatives and programs to promote improved knowledge, attitudes, and practices and reduce the effect of the risk factors contributing to CRC.

## 1 Introduction

Diet and lifestyle play fundamental roles in the prevention and management of numerous diseases, including colorectal cancer (CRC) (1–4). Globally, CRC is the third most common cancer and the second highest cause of cancer-related mortality (5). In Saudi Arabia, CRC is the most common cancer in men and the second most common cancer in women (6). Moreover, early-onset CRC, defined as that occurring in persons < 50 years of age, appears to be increasing in Saudi Arabia, and nearly one-third of individuals diagnosed with CRC are at an advanced disease stage at the time of diagnosis (7). This rising incidence of CRC among younger individuals coupled with the high proportion of end-stage cases is expected to increase the disease burden, leading to additional health complications that adversely affect the well-being of patients and further strain the healthcare system. It is essential to highlight the importance of studying early-onset CRC, as it helps develop targeted interventions and public health strategies that could tackle this concerning trend.

Even though the etiology of CRC is not entirely understood, genetics, aging, and, most importantly, poor diet and lifestyle factors such as smoking, physical inactivity, and obesity have been strongly linked with CRC risk (8). It has been estimated that dietary factors account for 70–90% of all CRC cases (9). Foods that are rich in fiber, antioxidants, probiotics, or polyphenols, such as fruit and vegetables, whole grains, and dairy products, have been reported to protect against CRC (10). By contrast, diets high in sugar, refined carbohydrates, fats, and protein, such as those based on processed meats and saturated/animal fats, have been estimated to contribute to nearly 80% of colon cancer cases (11).

In recent years, technological advances and the influence of the media have reshaped public knowledge, attitudes, and practices (KAP) toward various aspects of life, including the relationships between dietary and lifestyle choices and CRC development and prevention (12, 13). However, previous studies on public KAP levels toward dietary and lifestyle-related risk factors for CRC remain limited both at the global level and in Saudi Arabia.

Some research has been conducted to investigate the knowledge, beliefs, attitudes, and/or practices toward dietary and lifestyle-related risk factors for CRC. These studies were performed in various countries, including the United Kingdom (14), the United Arab Emirates (UAE) (15), Italy (16), Malaysia (17), Kuwait (18), and Lebanon (19), and indicated an overall lack of knowledge regarding CRC risk factors. However, these reports are now somewhat outdated, and their data cannot be considered entirely relevant to the current situation. Several studies were performed more recently in Jordan (20), the UAE (21), and Syria (22), which suggested good understanding but poor practices in relation to CRC risk factors that contribute to the development of the disease.

In Saudi Arabia, despite the concerns that the majority of CRC patients are diagnosed at advanced stages with metastases and over 85% of the Saudi population are < 50 years old and therefore at heightened risk of early-onset CRC, public KAP levels toward dietary and lifestyle-related risk factors for CRC are not well understood (7, 23). The majority of available studies focused on KAP assessment with respect to CRC bowel screening, signs, and symptoms (24–28). Therefore, the aim of the present work was to evaluate the KAP levels in terms of dietary and lifestyle-related risk factors for CRC and examine possible associations between the KAP scores and studied variables among the Saudi population. Such assessment is critical because the resulting knowledge and data would supply healthcare providers with the understanding necessary to implement appropriate public health strategies and educational programs to promote dietary habit and lifestyle modifications for reducing the risk of CRC and supporting better public health.

## 2 Materials and methods

### 2.1 Study design and participants

This cross-sectional survey was conducted between June and December 2023. The Biomedical Ethics Research Committee at King Abdulaziz University (Jeddah, Saudi Arabia) approved the study (Reference No. 97-23), and all of the participants provided informed consent. A convenience sample of 1,040 individuals aged 18 years or older participated in the study. Participants were asked to complete an online questionnaire prepared using Google Forms, ensuring that all questions were required to be answered, which resulting in no missing data. The questionnaire circulated using the snowball sampling technique via social media platforms such as WhatsApp and X (formerly known as Twitter). The sample size was calculated with the Epi Info online sample size calculator developed by the Division of Health Informatics and Surveillance of the Center for Surveillance, Epidemiology, and Laboratory Services of the U.S. Centers for Disease Control and Prevention (29) using data obtained from the Saudi General Authority for Statistics (58), including an estimated total population of 22,925,763 (≥18 years old). This calculation indicated that the effective sample size for this study was *n* = 664, with a 99% confidence interval and hypothesized 50% frequency of outcome factor in the population.

### 2.2 Study measures

The present study questionnaire was constructed following an extensive literature review (17, 30) to assess KAP levels toward dietary factors and the risk of CRC. The original questionnaire was developed in English and then translated into Arabic using the Brislin backtranslation method (31, 32). For pre-testing, the questionnaire was reviewed by five experts in clinical nutrition (PhD holders). The experts were asked to provide feedback regarding the questionnaire clarity, design, navigation difficulty, and ease of understanding the questions and potential answers. Some of the questions and answers were then revised based on the comments provided by the experts. The final version of the questionnaire consisted of four sections with a total of 46 questions in addition to the food frequency questionnaire and required ~20 min to complete. All study information, including the aims, inclusion criteria, estimated time to complete the questionnaire, and data confidentiality, were provided at the start of the questionnaire.

The first section of the questionnaire contained 20 questions regarding the sociodemographic and background characteristics of the participants, such as age, sex, marital status, educational level, work status, field of study, income, smoking habits, details of any chronic diseases, physical activity level, food allergies, primary source of medical and dietary information, and smoking habits. The self-reported height in centimeters and weight in kilograms were also used to compute the body mass index (BMI). In addition, two of the questions asked whether the participants or any of their first/second-degree relatives (parents, siblings, children, aunts/uncles, etc.) had been diagnosed with benign tumors (polyps) in the colon or CRC.

The second section included 14 questions assessing the participants' knowledge of dietary and lifestyle-related factors that may increase the risk of CRC according to international and local recommendations and guidelines. For example, these factors included dietary habits, weight, age, sex, family history, physical activity level, smoking habits, supplements use, taking aspirin and having certain health conditions. Participants were asked to select answers from a five-point Likert scale, with the possible responses ranging from 1 to 5 (1 = “strongly disagree”, 2 = “disagree”, 3 = “not sure”, 4 = “agree”, and 5 = “strongly agree”) for the correct statements, and reverse coding was applied for the incorrect statements. Next, the scores were classified into two categories, namely, low knowledge (below the third quartile: < 75th percentile; score < 52) and high knowledge (above the third quartile: >75th percentile; score > 52). The maximum score for the knowledge section was 70.

The third section contained 12 questions evaluating attitudes toward dietary and lifestyle-related factors that may increase or decrease the risk of CRC. The questions in this section asked the participants about their stances on the following factors for preventing CRC: dietary modification, consuming more dietary fiber, reducing consumption of low-fat foods, reducing consumption of foods high in sugar, exercising, avoiding smoking, reading nutritional information, and consulting a nutritionist. Participants were asked to select answers from a five-point Likert scale, with the possible responses ranging from 1 to 5 (1 = “strongly disagree”, 2 = “disagree”, 3 = “neutral”, 4 = “agree”, and 5 = “strongly agree”) for the positive attitude statements, and reverse coding was applied for the negative attitude statements. Next, the scores were classified into two categories, namely, negative attitudes (below the third quartile: < 75th percentile; score < 45) and positive attitudes (above the third quartile: >75th percentile; score > 45). The maximum score for the attitudes section was 60.

In the fourth section, the dietary practices of participants were assessed using a food frequency questionnaire, in which participants stated how often they consumed each of 31 food items that have been reported to have positive or negative associations with CRC, such as vegetables, fruits, processed meat, red meat, fish, and dairy products. For each food item, participants were asked to report their frequency of intake in the last 6 months on a nine-point Likert scale, with the possible responses ranging from 1 to 9 (1 = “less than once per month/never”, 2 = “1–3 times per month”, 3 = “once per week”, 4 = “2–4 times per week”, 5 = “5–6 times per week”, 6 = “once per day”, 7 = “2–3 times per day”, 8 = “4–5 times per day”, and 9 = “6+ times per day”) for the good practices, and reverse coding was applied for the poor practices. Next, the scores were classified into two categories, namely, poor practices (below the third quartile: < 75th percentile; score < 211) and good practices (above the third quartile: >75th percentile; score > 211). The maximum score for the practices section was 279.

### 2.3 Statistical analysis

The IBM SPSS software program (version 25, SPSS Inc., Chicago, IL, USA) was used for statistical analysis. Descriptive statistics including numbers and percentages were calculated for qualitative variables, while the means, standard deviations, medians, and interquartile ranges (IQRs) were calculated for quantitative variables. Normality tests were used to determine the normality of quantitative variables. The KAP scores were divided into two categories as per Bloom's criteria: low vs. high knowledge, positive vs. negative attitudes, and good vs. poor practices. Chi-square tests were used to find the association between qualitative variables. Binary logistic regression models were used to find the predictors of KAP scores in terms of dietary and lifestyle-related risk factors for CRC among the study participants. A *p*-value of < 0.05 was considered to be statistically significant.

## 3 Results

### 3.1 Sample characteristics

A total of 1,040 participants were involved in the study. Table 1 presents their sociodemographic and background characteristics. The participants displayed relatively even distributions of both age (18–24 years: 29.0%; 25–39 years: 35.4%; 40–59 years: 30.2%; ≥60 years: 5.4%) and sex (male: 45.1%; female: 54.9%). Just over half of participants (53.3%) reported being married, while 43.6% were single and the remainder were divorced or widowed. Over half (58.2%) reported having a university-level education, and almost half (45.4%) were employed. A similar proportion (44.4%) reported their field of study as scientific, and over one-third (37.8%) reported earning more than 10,000 Saudi riyals (>2,665 U.S. dollars) per month. The calculated BMI values indicated that 8.3% of participants were underweight, 35.4% were of normal weight, 29.9% were overweight, and 26.4% were obese. The majority of participants reported being either very active (22.4%) or moderately active (37.4%), while 40.2% were fairly inactive. Most participants reported being non-smokers (79.5%), having no history of chronic diseases (78.2%), having no food allergies (84.3%), and using social media as their primary source of dietary information (56.9%). Only 8.4% of participants reported having personal or familial experience with CRC or polyps. Only 0.3% of participants have been personally diagnosed benign tumors (polyps) in the colon, while 8.1% reported having first/second-degree relatives diagnosed with colon polyps. Similarly, 0.4% of participants have been personally diagnosed CRC, while 8.4% reported having first/second-degree relatives diagnosed with CRC.

**Table 1:** Sociodemographic and background characteristics of the participants (*n* = 1,040).

**Variable**	**Frequency (*n*)**	**Percentage (%)**
**Age (years)**		
18–24	302	29.0
25–39	368	35.4
40–59	314	30.2
≥60	56	5.4
**Sex**		
Male	469	45.1
Female	571	54.9
**Marital status**		
Single	453	43.6
Married	554	53.3
Divorced	25	2.4
Widowed	8	0.7
**Educational level**		
Less than high school	39	3.8
High school	200	19.2
University	605	58.2
Postgraduate	196	18.8
**Work status**		
Student	281	27.0
Employed	472	45.4
Unemployed	169	16.2
Retired	81	7.8
Business/trading	37	3.6
**Field of study**		
Medical	237	22.8
Scientific	462	44.4
Literature	270	26.0
No specific field	71	6.8
**Income (Saudi riyals per month)**		
No income	175	16.8
< 2,000	180	17.3
2,000–4,999	111	10.7
5,000–7,999	82	7.9
8,000–10,000	99	9.5
>10,000	393	37.8
**BMI** ^*^		
Underweight	86	8.3
Normal	368	35.4
Overweight	311	29.9
Obese	275	26.4
No	827	79.5
Yes	168	16.2
Ex-smoker	45	4.3
**History of chronic diseases**		
No	813	78.2
Yes	227	21.8
**Physical activity**		
Fairly inactive	418	40.2
Moderately active	389	37.4
Very active	233	22.4
**Food allergies**		
No	877	84.3
Yes	163	15.7
**Primary source of medical/dietary information**		
Family members	161	15.5
Friends/peers/colleagues	31	3.0
Books/magazines	8	0.8
Internet/website	94	9.0
Media (TV, radio)	19	1.8
Social media	592	56.9
Healthcare professionals	114	11.0
Organizations	21	2.0
**Have you or one of your first/second-degree relatives**		
**(parents, siblings, children, aunts/uncles, etc.) been diagnosed**		
**with benign tumors (polyps) in the colon?**		
I have not been diagnosed with polyps	461	44.3
None of my relatives have been diagnosed with polyps	492	47.3
I have been diagnosed with polyps	3	0.3
One of my relatives has been diagnosed with polyps	84	8.1
**Have you or one of your first/second-degree relatives (parents**,		
**siblings, children, aunts/uncles, etc.) been diagnosed**		
**with CRC?**		
I have not been diagnosed with CRC	440	42.3
None of my relatives have been diagnosed with CRC	508	48.9
I have been diagnosed with CRC	4	0.4
One of my relatives has been diagnosed with CRC	88	8.4

^*^Calculated based on self-reported weight and height.

### 3.2 Distribution of KAP scores

Table 2 presents the classification results for the KAP scores of the participants with respect to the dietary and lifestyle-related risk factors for CRC. The data revealed that a majority of participants (77.8%) had low knowledge about these risk factors for CRC, while only 22.2% possessed high knowledge. Similarly, 78.6% of participants displayed negative attitudes toward these risk factors, with just 21.4% having positive attitudes. In terms of practices, 75.0% of participants were found to engage in poor practices relating to CRC risk factors, leaving only 25.0% demonstrating good practices. The median (IQR) KAP scores for knowledge, attitudes, and practices were 48 (43–53), 48 (44–53), and 165 (154–176.7), respectively.

**Table 2:** Classification results for the KAP scores of the participants with respect to the risk factors for CRC.

**Variable**	**Classification**	***n* (%)**
Knowledge^a^	Low	809 (77.8)
	High	231 (22.2)
Attitudes^b^	Negative	817 (78.6)
	Positive	223 (21.4)
Practices^c^	Poor	780 (75.0)
	Good	260 (25.0)

^a^Low knowledge was defined as scores below the 75th percentile, corresponding to < 52. ^b^Negative attitudes were defined as scores below the 75th percentile, corresponding to < 45. ^c^Poor practices were defined as scores below the 75th percentile, corresponding to < 211.

### 3.3 Knowledge of dietary and lifestyle-related risk factors for CRC

Table 3 presents the knowledge levels of the participants with respect to dietary and lifestyle-related risk factors for CRC, according to international and local recommendations and guidelines. These were categorized into “low knowledge” and “high knowledge”, and an overall knowledge score was also calculated. Participants displayed low knowledge about daily intake aspirin (84.2%), consuming fewer than five servings of fruits and vegetables per day (66.5%), and daily supplement intake (68.1%). By contrast, more participants had high knowledge about the risk factors of smoking (65.6%), consuming processed meat once per day or more (66.2%) and being overweight or obese (defined as a BMI of over 25; 62.9%). Participants were evenly split in their knowledge of the risk factor of being older than 50 years, where 50.9% had low knowledge and 49.1% had high knowledge. In terms of overall knowledge, the minimum and maximum scores were 22 and 68, respectively, with a median score of 48 (IQR: 43–53). The mean score was 48.04 (SD: 7.31).

**Table 3:** Knowledge levels of the participants about dietary and lifestyle-related risk factors for CRC (*n* = 1,040).

**Knowledge questions of CRC risk factors according to international and local recommendations and guidelines**	Possible range^*^	**Low knowledge *n* (%)**	**High knowledge *n* (%)**
1. Consuming fewer than five servings of fruits and vegetables per day	1–5	692 (66.5)	348 (33.5)
2. Consuming red meat once per day or more	1–5	664 (63.8)	376 (36.2)
3. Consuming processed meat once per day or more	1–5	352 (33.8)	688 (66.2)
4. Following a diet low in dietary fiber	1–5	473 (45.5)	567 (54.5)
5. Overweight or obesity (BMI > 25)	1–5	386 (37.1)	654 (62.9)
6. Age over 50 years	1–5	529 (50.9)	511 (49.1)
7. Having first/second-degree relatives with colorectal cancer	1–5	468 (45.0)	572 (55.0)
8. Physical activity fewer than 150 min per week	1–5	564 (54.2)	476 (45.8)
9. Having digestive diseases (such as Crohn's disease and ulcerative colitis)	1–5	438 (42.1)	602 (57.9)
10. Having diabetes	1–5	675 (64.9)	365 (35.1)
11. Smoking	1–5	358 (34.4)	682 (65.6)
12. Daily intake of aspirin	5–1	876 (84.2)	164 (15.8)
13. Daily intake of supplements (multivitamins, minerals, and herbs)	5–1	708 (68.1)	332 (31.9)
14. Men are more susceptible than women	1–5	666 (64.0)	374 (36.0)
**Knowledge score**			
Minimum score		22	
Maximum score		68	
Median (IQR)		48 (43–53)	
Mean (SD)		48.0 (7.3)	

^*^The possible range of 1–5 represents responses from 1 for “strongly disagree” to 5 for “strongly agree” for correct statements, and reverse coding was applied for the incorrect statements. A high knowledge score means that the respondent was considered to have high knowledge, whereas a low knowledge score means that the respondent was considered to have low knowledge.

### 3.4 Attitudes toward dietary and lifestyle-related risk factors for CRC

Table 4 presents the attitudes of the participants toward dietary and lifestyle-related risk factors for CRC. These were categorized into “negative attitudes” and “positive attitudes”, and an overall attitude score was also calculated. Among the participants, 43.4% felt that they did not need to modify their diet owing to a low perceived risk, while 56.6% believed that they should modify their diet for CRC prevention. A majority (72.3%) recognized the importance of them following a healthy diet, and 84.6% agreed that they should maintain a healthy weight to lower their CRC risk. Increasing their dietary fiber consumption was regarded as important by 83.7% of participants, whereas reducing their intake of low-fat foods was considered less of a priority with only 21.1% in agreement. Most participants (85.1%) acknowledged the importance of reducing their consumption of high-sugar foods. A large majority (88.5%) agreed with the importance of being aware of global dietary recommendations for preventing CRC, and 78.3% agreed with the importance of eating more green vegetables and reducing red meat consumption. Most participants (85.1%) also recognized that they should exercise to reduce their risk of developing CRC, and 78.4% were aware of the link between smoking and CRC risk. Most participants (67.0%) reported reading the nutrition information on products, and 81.7% agreed that they should consult a nutritionist when needed. In terms of the overall attitude score, the minimum and maximum scores were 20 and 60, respectively, with a median score of 48 (IQR: 44–53). The mean score was 47.83 (SD: 6.59).

**Table 4:** Attitudes of the participants toward dietary and lifestyle-related risk factors for CRC (*n* = 1,040).

**What is your stance on the following factors for prevention of CRC?**	Possible range^*^	**Negative attitude *n* (%)**	**Positive attitude *n* (%)**
1. No, I don't need to modify my diet as I'm not at risk for CRC.	5–1	451 (43.4)	589 (56.6)
2. I don't want to follow a healthy diet as it is not worthwhile for preventing CRC.	5–1	288 (27.7)	752 (72.3)
3. I should maintain a healthy weight to reduce the risk of colorectal cancer.	1–5	160 (15.4)	880 (84.6)
4. I should increase my consumption of dietary fiber.	1–5	170 (16.3)	870 (83.7)
5. I should increase my intake of high-fat foods.	5–1	219 (21.1)	821 (78.9)
6. I should reduce my intake of high-sugar foods.	1–5	155 (14.9)	885 (85.1)
7. It is important to know the global dietary recommendations for preventing CRC.	1–5	120 (11.5)	920 (88.5)
8. It is important to eat more green vegetables and reduce red meat consumption to protect against CRC.	1–5	226 (21.7)	814 (78.3)
9. I should exercise to reduce the risk of developing the disease.	1–5	155 (14.9)	885 (85.1)
10. I know that smoking can increase the risk.	1–5	225 (21.6)	815 (78.4)
11. I read the nutrition information on products.	1–5	343 (33.0)	697 (67.0)
12. I should consult a nutritionist when needed.	1–5	190 (18.3)	850 (81.7)
**Attitude score**			
Minimum score		20	
Maximum score		60	
Median (IQR)		48 (44–53)	
Mean (SD)		47.8 (6.5)	

^*^The possible range of 1–5 represents responses from 1 for “strongly disagree” to 5 for “strongly agree” for the positive attitude statements, and reverse coding was applied for the negative attitude statements. A high attitude score means that the respondent was considered to have positive attitudes toward CRC risk factors, whereas a low attitude score means that the respondent was considered to have negative attitudes toward CRC risk factors.

### 3.5 Practices to address dietary and lifestyle-related risk factors for CRC

Table 5 presents the dietary practices reported by the participants regarding selected food items. These were categorized into “poor practices” and “good practices”, and an overall practice score was also calculated. High proportions of participants demonstrated good practices toward the consumption of canned fruit (72.9%), fresh vegetables (71.2%), and processed meats (70.2%). However, 79.8% of participants displayed the poor practices of consuming starches such as white bread, rice, pasta, and pastries, as well as dried fruit (62.6%) and milk alternatives (63.2%). In terms of the overall practice score, the minimum and maximum scores were 111 and 238, respectively, with a median score of 165 (IQR: 154–176.75). The mean score was 165.70 (SD: 17.85).

**Table 5:** Practices of the participants toward dietary and lifestyle-related risk factors for CRC (*n* = 1,040).

**Food item**	Possible range^*^	**Poor practice *n* (%)**	**Good practice *n* (%)**
1. Fresh fruit	9–1	334 (32.1)	706 (67.9)
2. Dried fruit	9–1	651 (62.6)	389 (37.4)
3. Canned fruit	1–9	282 (27.1)	758 (72.9)
4. Fresh fruit juice	9–1	563 (54.1)	477 (45.9)
5. Juices with added sugar	1–9	338 (32.5)	702 (67.5)
6. Fresh vegetables	9–1	300 (28.8)	740 (71.2)
7. Leafy greens	9–1	319 (30.7)	721 (69.3)
8. Cooked vegetables	9–1	363 (34.9)	677 (65.1)
9. Legumes	9–1	519 (49.9)	521 (50.1)
10. Salted nuts	1–9	568 (54.6)	472 (45.4)
11. Seeds	9–1	628 (60.4)	412 (39.6)
12. Starches (white bread, rice, pasta, pastries, etc.)	1–9	830 (79.8)	210 (20.2)
13. Whole grains (whole wheat bread, whole wheat pasta, bulgur, etc.)	9–1	365 (35.1)	675 (64.9)
14. Breakfast cereals	9–1	651 (62.6)	389 (37.4)
15. Red meat	1–9	572 (55.0)	468 (45.0)
16. Processed meats	1–9	310 (29.8)	730 (70.2)
17. White meat	9–1	369 (35.5)	671 (64.5)
18. Processed white meat	1–9	748 (71.9)	292 (28.1)
19. Fish/seafood	9–1	605 (58.2)	435 (41.8)
20. Tuna	9–1	632 (60.8)	408 (39.2)
21. Non-meat protein (eggs, tofu, quinoa, etc.)	9–1	392 (37.7)	648 (62.3)
22. Fast food (burgers, fries, etc.)	1–9	405 (38.9)	635 (61.1)
23. Low-fat dairy products (milk, cheese, yogurt, etc.)	9–1	464 (44.6)	576 (55.4)
24. Full-fat dairy products (milk, cheese, yogurt, etc.)	1–9	683 (65.7)	357 (34.3)
25. Carbonated beverages	1–9	369 (35.5)	671 (64.5)
26. Carbonated beverages without sugar	1–9	367 (35.3)	673 (64.7)
27. Sweets (Western sweets, Eastern sweets, etc.)	1–9	454 (43.7)	586 (56.3)
28. Canned/tinned foods	1–9	363 (34.9)	677 (65.1)
29. Milk alternatives (rice, oat, soy, coconut, etc.)	9–1	657 (63.2)	383 (36.8)
30. Animal fats (ghee, butter, etc.)	1–9	506 (48.7)	534 (51.3)
31. Vegetable oils (olive, flaxseed, sesame, avocado, etc.)	9–1	405 (38.9)	635 (61.1)
**Practice score**			
Minimum score		111	
Maximum score		238	
Median (IQR)		165 (154–176.7)	
Mean (SD)		165.7 (17.8)	

^*^The possible range of 1–9 represents responses ranging from 1 for a consumption frequency of “less than once per month/never” to 9 for a consumption frequency of “6+ times per day” for the good practices, and reverse coding was applied for the poor practices. A high practice score means that the respondent was considered to have good practices, whereas a low practice score means that the respondent was considered to have poor practices.

### 3.6 Associations between the study variables and KAP scores

Table 6 presents the associations between the study variables and KAP scores with respect to dietary and lifestyle-related risk factors for CRC. The results indicated that the age did not show a significant different in knowledge, however, a more positive attitude score was found among younger groups compared with the older age groups (*p* = 0.008). However, practice score was higher among older groups compared to the younger (*p* = 0.001). Sex differences were also evident, with males showing lower knowledge scores than females (*p* = 0.034), although the associations with the attitude and practice scores were not as strong. Single individuals exhibited higher knowledge scores (*p* = 0.010) compared with married, divorced, and widowed individuals. However, married participants found to have higher practice score (*p* = 0.001). Higher educational levels were not significantly correlated with better knowledge, attitude, or practice scores. Participants from medical background showed higher knowledge (*p* = 0.001), with no associations with the attitude and practice scores. Individuals who received medical and dietary information from media (TV and radio) and organization tended to exhibit higher knowledge (*p* = 0.008) and attitude scores (*p* = 0.001). Lastly, having a personal or one of first/second-degree relatives been diagnosed with polyps significantly affected the knowledge (*p* = 0.015), attitude (*p* = 0.004), and practice scores (*p* = 0.001). similarly, having a personal or one of first/second-degree relatives been diagnosed with CRC significantly affected the knowledge (*p* = 0.041), attitude (*p* = 0.003), and practice scores (*p* = 0.011).

**Table 6:** Associations between the study variables and KAP scores with respect to dietary and lifestyle-related risk factors for CRC (*n* = 1,040).

**Variable**	**Knowledge score**			**Attitude score**			**Practice score**		
	**Low knowledge**	**High knowledge**	***p*** **-value**	**Negative attitude**	**Positive attitude**	***p*** **-value**	**Poor practice**	**Good practice**	***p*** **-value**
	***n*** **(%)**	***n*** **(%)**		***n*** **(%)**	***n*** **(%)**		***n*** **(%)**	***n*** **(%)**	
**Age (years)**									
18–24	227 (75.2)	75 (24.8)	0.052	227 (75.2)	75 (24.8)	0.008^*^	237 (78.5)	65 (21.5)	0.001^*^
25–39	277 (75.3)	91 (24.7)		278 (75.5)	90 (24.5)		292 (79.3)	76 (20.7)	
40–59	261 (83.1)	53 (16.9)		263 (83.8)	51 (16.2)		213 (67.8)	101 (32.2)	
≥60	44 (78.6)	12 (21.4)		49 (87.5)	7 (12.5)		38 (67.9)	18 (32.1)	
**Sex**									
Male	379 (80.8)	90 (19.2)	0.034^*^	380 (81)	89 (19)	0.079	363 (77.4)	106 (22.6)	0.105
Female	430 (75.3)	141 (24.7)		437 (76.5)	134 (23.5)		417 (73)	154 (27)	
**Marital status**									
Single	332 (73.3)	121 (26.7)	0.010^*^	344 (75.9)	109 (24.1)	0.202	364 (80.4)	89 (19.6)	0.001^*^
Married	447 (80.7)	107 (19.3)		448 (80.9)	106 (19.1)		387 (69.9)	167 (30.1)	
Divorced	23 (92)	2 (8)		18 (72)	7 (28)		23 (92)	2 (8)	
Widowed	7 (87.5)	1 (12.5)		7 (87.5)	1 (12.5)		6 (75)	2 (25)	
**Educational level**									
Less than high school	34 (87.2)	5 (12.8)	0.45	29 (74.4)	10 (25.6)	0.633	26 (66.7)	13 (33.3)	0.629
High school	157 (78.5)	43 (21.5)		163 (81.5)	37 (18.5)		148 (74)	52 (26)	
University	470 (77.7)	135 (22.3)		474 (78.3)	131 (21.7)		458 (75.7)	147 (24.3)	
Postgraduate	148 (75.5)	48 (24.5)		151 (77)	45 (23)		148 (75.5)	48 (24.5)	
**Work status**									
Student	202 (71.9)	79 (28.1)	0.068	216 (76.9)	65 (23.1)	0.088	230 (81.9)	51 (18.1)	0.037^*^
Employed	377 (79.9)	95 (20.1)		379 (80.3)	93 (19.7)		338 (71.6)	134 (28.4)	
Unemployed	133 (78.7)	36 (21.3)		122 (72.2)	47 (27.8)		125 (74)	44 (26)	
Retired	68 (84)	13 (16)		69 (85.2)	12 (14.8)		59 (72.8)	22 (27.2)	
Business/trading	29 (78.4)	8 (21.6)		31 (83.8)	6 (16.2)		28 (75.7)	9 (24.3)	
**Field of study**									
Medical	149 (62.9)	88 (37.1)	0.001^*^	177 (74.7)	60 (25.3)	0.278	183 (77.2)	54 (22.8)	0.067
Scientific	377 (81.6)	85 (18.4)		363 (78.6)	99 (21.4)		355 (76.8)	107 (23.2)	
Literature	224 (83)	46 (17)		221 (81.9)	49 (18.1)		197 (73)	73 (27)	
No specific field	59 (83.1)	12 (16.9)		56 (78.9)	15 (21.1)		45 (63.4)	26 (36.6)	
**Income (Saudi riyals per month)**									
No income	135 (77.1)	40 (22.9)	0.635	135 (77.1)	40 (22.9)	0.288	147 (84)	28 (16)	0.035^*^
< 2,000	136 (75.6)	44 (24.4)		140 (77.8)	40 (22.2)		138 (76.7)	42 (23.3)	
2,000–4,999	81 (73)	30 (27)		80 (72.1)	31 (27.9)		82 (73.9)	29 (26.1)	
5,000–7,999	67 (81.7)	15 (18.3)		65 (79.3)	17 (20.7)		59 (72)	23 (28)	
8,000–10,000	79 (79.8)	20 (20.2)		75 (75.8)	24 (24.2)		67 (67.7)	32 (32.3)	
>10,000	311 (79.1)	82 (22.2)		322 (81.9)	71 (18.1)		287 (73)	106 (27)	
**BMI**									
Underweight	71 (82.6)	15 (17.4)	0.645	65 (75.6)	21 (24.4)	0.846	71 (82.6)	15 (17.4)	0.149
Normal	281 (76.4)	87 (23.6)		290 (78.8)	78 (21.2)		283 (76.9)	85 (23.1)	
Overweight	244 (78.5)	67 (21.5)		248 (79.7)	63 (20.3)		223 (71.7)	88 (28.3)	
Obese	213 (77.5)	62 (22.5)		214 (77.8)	61 (22.2)		203 (73.8)	72 (26.2)	
**Smoking habits**									
No	634 (76.7)	193 (23.3)	0.209	637 (77)	190 (23)	0.055	618 (74.7)	209 (25.3)	0.018^*^
Yes	137 (81.5)	31 (18.5)		143 (85.1)	25 (14.9)		135 (80.4)	33 (19.6)	
Ex-smoker	38 (84.4)	7 (15.6)		37 (82.2)	8 (17.8)		27 (60)	18 (40)	
**History of chronic diseases**									
No	628 (77.2)	185 (22.8)	0.425	638 (78.5)	175 (21.5)	0.902	617 (75.9)	196 (24.1)	0.209
Yes	181 (79.7)	46 (20.3)		179 (78.9)	48 (21.1)		163 (71.8)	64 (28.2)	
**Physical activity**									
Fairly inactive	336 (80.4)	82 (19.6)	0.24	341 (81.6)	77 (18.4)	0.037^*^	337 (80.6)	81 (19.4)	0.003^*^
Moderately active	294 (75.6)	95 (24.4)		306 (78.7)	83 (21.3)		278 (71.5)	111 (28.5)	
Very active	179 (76.8)	54 (23.2)		170 (73)	63 (27)		165 (70.8)	68 (29.2)	
**Food allergies**									
No	688 (78.4)	189 (21.6)	0.234	696 (79.4)	181 (20.6)	0.143	647 (73.8)	230 (26.2)	0.034^*^
Yes	121 (74.2)	42 (25.8)		121 (74.2)	42 (25.8)		133 (81.6)	30 (18.4)	
**Primary source of medical/dietary information**									
Family members	139 (86.3)	22 (13.7)	0.008^*^	146 (90.7)	15 (9.3)	0.001^*^	120 (74.5)	41 (25.5)	0.184
Friends/peers/colleagues	24 (77.4)	7 (22.6)		27 (87.1)	4 (12.9)		25 (80.6)	6 (19.4)	
Books/magazines	8 (100)	0 (0)		8 (100)	0 (0)		6 (75)	2 (25)	
Internet/website	77 (81.9)	17 (18.1)		79 (84)	15 (16)		70 (74.5)	24 (25.5)	
Media (TV, radio)	12 (63.2)	7 (36.8)		11 (57.9)	8 (42.1)		14 (73.7)	5 (26.3)	
Social media	453 (76.5)	139 (23.5)		446 (75.3)	146 (24.7)		454 (76.7)	138 (23.3)	
Healthcare professionals	84 (73.7)	30 (26.3)		85 (74.6)	29 (25.4)		73 (64)	41 (36)	
Organizations	12 (57.1)	9 (42.9)		15 (71.4)	6 (28.6)		18 (85.7)	3 (14.3)	
**Have you or one of your first/second-degree relatives (parents, siblings, children, aunts/uncles, etc.) been diagnosed with benign**									
**tumors (polyps) in the colon?**									
I have not been diagnosed with polyps	365 (79.2)	96 (20.8)	0.015^*^	381 (82.6)	80 (17.4)	0.004^*^	317 (68.8)	144 (31.2)	0.001^*^
None of my relatives have been diagnosed with polyps	387 (78.7)	105 (21.3)		377 (76.6)	115 (23.4)		399 (81.1)	93 (18.9)	
I have been diagnosed with polyps	3 (100)	0 (0)		3 (100)	0 (0)		3 (100)	0 (0)	
One of my relatives has been diagnosed with polyps	54 (64.3)	30 (35.7)		56 (66.7)	28 (33.3)		61 (72.6)	23 (27.4)	
**Have you or one of your first/second-degree relatives (parents, siblings, children, aunts/uncles, etc.) been diagnosed with CRC?**									
I have not been diagnosed with CRC	351 (79.8)	89 (20.2)	0.041^*^	366 (83.2)	74 (16.8)	0.003^*^	309 (70.2)	131 (29.8)	0.011^*^
None of my relatives have been diagnosed with CRC	397 (78.1)	111 (21.9)		387 (76.2)	121 (23.8)		404 (79.5)	104 (20.5)	
I have been diagnosed with CRC	3 (75)	1 (25)		4 (100)	0 (0)		3 (75)	1 (25)	
One of my relatives has been diagnosed with CRC	58 (65.9)	30 (34.1)		60 (68.2)	28 (31.8)		64 (72.7)	24 (27.3)	

^*^Significant *p* value.

Table 7 presents the results of binary logistic regression models assessing the potential determinants of KAP scores with respect to the dietary and lifestyle-related risk factors for CRC. Sex analysis revealed that female participants were more likely to have better dietary practices related to CRC risk factors compared with males (*p* = 0.001). However, no significant gender differences were observed in the knowledge and attitude scores. Interestingly, participants in the field of literature exhibited significantly (*p* = 0.001) higher odds of having higher knowledge compared with those in the medical field, while those with no specific field of study had significantly (*p* = 0.008) higher practice scores compared with those in the medical field. Smokers showed significantly (*p* = 0.002) higher odds of higher practice scores compared with non-smokers, but smoking did not significantly affect the knowledge or attitude scores. Physical activity level played a significant role, with moderately active participants showing significantly higher attitude (*p* = 0.006) and practice scores (*p* = 0.005) compared with those who were fairly inactive. Very active participants also exhibited higher attitude scores (*p* = 0.023). Individuals with food allergies had significantly higher odds of higher practice scores (*p* = 0.033). Participants who primarily received their medical and dietary information from friends, peers, or colleagues had significantly (*p* = 0.047) lower knowledge and attitude scores (*p* = 0.032) compared with those who received information from family members. By contrast, those who received information from organizations displayed significantly higher practice scores (*p* = 0.044). Having a first/second-degree relatives diagnosed polyps or CRC did not significantly influence knowledge, attitude, or practice scores.

**Table 7:** Binary logistic regression model results for potential determinants of KAP scores with respect to the dietary and lifestyle-related risk factors for CRC.

**Variable**	**Knowledge score**		**Attitude score**		**Practice score**	
	**OR (95% CI)**	***p*-value**	**OR (95% CI)**	***p*-value**	**OR (95% CI)**	***p*-value**
**Age (years)**						
18–24	1		1		1	
25–39	0.363 (0.107–1.227)	0.103	3.024 (0.818–11.183)	0.097	1.035 (0.344–3.114)	0.951
40–59	0.600 (0.204–1.769)	0.355	2.203 (0.679–7.150)	0.189	0.551 (0.219–1.390)	0.207
≥60	0.522 (0.189–1.439)	0.209	1.418 (0.470–4.278)	0.535	0.739 (0.318–1.721)	0.483
**Sex**						
Male	1		1		1	
Female	1.366 (0.916–2.037)	0.126	0.892 (0.596–1.333)	0.576	1.990 (1.342–2.950)	0.001^*^
**Marital status**						
Single	1		1		1	
Married	0.452 (0.033–6.223)	0.553	1.443 (0.118–17.682)	0.774	0.376 (0.038–3.696)	0.402
Divorced	1.135 (0.129–9.988)	0.909	1.102 (0.107–11.359)	0.935	2.542 (0.447–14.452)	0.293
Widowed	1.552 (0.168–14.321)	0.698	1.019 (0.094–10.999)	0.988	1.678 (0.276–10.193)	0.574
**Educational level**						
Less than high school	1		1		1	
High school	0.928 (0.598–1.440)	0.74	0.787 (0.506–1.224)	0.287	0.997 (0.648–1.532)	0.988
University	0.893 (0.486–1.640)	0.714	0.720 (0.388–1.335)	0.297	1.284 (0.710–2.322)	0.409
Postgraduate	0.614 (0.192–1.703)	0.41	1.303 (0.483–3.511)	0.601	0.886 (0.324–2.420)	0.813
**Work status**						
Student	1		1		1	
Employed	0.689 (0.376–1.260)	0.226	0.611 (0.340–1.095)	0.098	1.052 (0.588–1.884)	0.864
Unemployed	0.886 (0.330–2.378)	0.811	0.552 (0.199–1.536)	0.255	0.839 (0.321–2.196)	0.721
Retired	0.572 (0.192–1.703)	0.316	0.652 (0.227–1.871)	0.427	0.563 (0.223–1.423)	0.225
Business/trading	1.610 (0.871–2.978)	0.129	0.663 (0.367–1.200)	0.175	0.562 (0.293–1.081)	0.084
**Field of study**						
Medical	1		1		1	
Scientific	0.888 (0.577–1.369)	0.592	0.798 (0.525–1.213)	0.292	0.919 (0.622–1.356)	0.67
Literature	2.431 (1.649–3.585)	0.001^*^	1.096 (0.732–1.642)	0.656	1.012 (0.666–1.535)	0.957
No specific field	1.045 (0.478–2.282)	0.912	1.016 (0.479–2.157)	0.967	2.547 (1.272–5.102)	0.008^*^
**Income (Saudi riyals per month)**						
No income	1		1		1	
< 2,000	1.317 (0.711–2.438)	0.381	1.412 (0.768–2.593)	0.267	1.050 (0.580–1.903)	0.871
2,000–4,999	0.878 (0.431–1.789)	0.72	1.140 (0.573–2.266)	0.709	1.108 (0.592–2.076)	0.749
5,000–7,999	0.966 (0.528–1.768)	0.91	1.516 (0.852–2.700)	0.157	1.206 (0.704–2.066)	0.495
8,000–10,000	0.790 (0.405–1.542)	0.491	0.845 (0.430–1.660)	0.626	1.114 (0.572–2.171)	0.751
>10,000	0.780 (0.395–1.540)	0.475	0.814 (0.412–1.608)	0.554	0.506 (0.250–1.026)	0.059
**BMI**						
Underweight	1		1		1	
Normal	0.509 (0.255–1.016)	0.055	0.862 (0.456–1.627)	0.646	0.672 (0.331–1.363)	0.271
Overweight	0.823 (0.538–1.258)	0.368	0.736 (0.480–1.128)	0.16	1.010 (0.663–1.539)	0.962
Obese	1.016 (0.664–1.556)	0.941	0.868 (0.567–1.330)	0.516	1.215 (0.813–1.815)	0.342
**Smoking**						
No	1		1		1	
Yes	0.777 (0.295–2.046)	0.609	1.054 (0.418–2.657)	0.912	3.323 (1.533–7.203)	0.002^*^
Ex-smoker	1.038 (0.631–1.709)	0.883	1.491 (0.882–2.522)	0.136	1.207 (0.744–1.957)	0.446
**History of chronic diseases**						
No	1		1		1	
Yes	1.002 (0.666–1.506)	0.993	0.893 (0.596–1.338)	0.583	0.829 (0.567–1.212)	0.333
**Physical activity**						
Fairly inactive	1		1		1	
Moderately active	0.686 (0.449–1.048)	0.082	0.562 (0.372–0.851)	0.006^*^	0.554 (0.368–0.833)	0.005^*^
Very active	0.923 (0.610–1.398)	0.706	0.623 (0.415–0.936)	0.023^*^	0.978 (0.661–1.447)	0.911
**Food allergies**						
No	1		1		1	
Yes	0.865 (0.571–1.310)	0.493	0.834 (0.552–1.260)	0.389	1.642 (1.041–2.590)	0.033^*^
**Primary source of medical/dietary information**						
Family members	1		1		1	
Friends/peers/colleagues	0.343 (0.119–0.986)	0.047^*^	0.283 (0.089–0.896)	0.032^*^	2.605 (0.677–10.021)	0.164
Books/magazines	0.637 (0.172–2.359)	0.5	0.494 (0.112–2.174)	0.351	1.972 (0.397–9.780)	0.406
Internet website	0.000 (0.000–0.000)	0.999	0.000 (0.000–0.000)	0.999	1.610 (0.184–14.114)	0.667
Media (TV, radio)	0.402 (0.136–1.187)	0.099	0.525 (0.167–1.655)	0.271	2.661 (0.677–10.457)	0.161
Social media	1.050 (0.272–4.049)	0.943	1.813 (0.460–7.148)	0.395	2.397 (0.450–12.763)	0.306
Healthcare professionals	0.538 (0.207–1.393)	0.201	0.779 (0.283–2.142)	0.628	2.781 (0.764–10.115)	0.121
Organizations	0.578 (0.205–1.628)	0.299	0.849 (0.285–2.528)	0.768	3.973 (1.037–15.223)	0.044^*^
**Have you or one of your first/second-degree relatives (parents, siblings, children, aunts/uncles, etc.) been diagnosed with**						
**benign tumors (polyps) in the colon?**						
I have not been diagnosed with polyps	1		1		1	
None of my relatives have been diagnosed with polyps	0.854 (0.389–1.876)	0.694	0.745 (0.335–1.659)	0.472	1.548 (0.714–3.356)	0.268
I have been diagnosed with polyps	0.762 (0.385–1.508)	0.435	0.750 (0.382–1.476)	0.405	0.587 (0.284–1.214)	0.151
One of my relatives has been diagnosed with polyps	0.000 (0.000–0.000)	0.999	0.000 (0.000–0.000)	0.999	0.000 (0.000–0.000)	0.999
**Have you or one of your first/second-degree relatives (parents, siblings, children, aunts/uncles, etc.) been diagnosed with CRC?**						
I have not been diagnosed with CRC	1		1		1	
None of my relatives have been diagnosed with CRC	0.632 (0.293–1.364)	0.242	0.529 (0.242–1.158)	0.111	0.795 (0.375–1.687)	0.551
I have been diagnosed with CRC	0.580 (0.293–1.149)	0.118	0.661 (0.336–1.298)	0.229	1.033 (0.502–2.125)	0.929
One of my relatives has been diagnosed with CRC	5.170 (0.274–97.568)	0.273	0.000 (0.000–0.000)	0.999	1.222 (0.067–22.307)	0.892

^*^Significant p-value.

Table 8 presents the correlation coefficients between knowledge, attitudes, and practices with respect to the dietary and lifestyle-related risk factors for CRC. The table lists the Spearman's rank correlation coefficients (ρ) and their corresponding *p*-values to assess the strength and significance of the relationships between the three domains. The correlation between knowledge and attitudes was found to be significant with a ρ value of 0.293 (*p* = 0.001), indicating a moderate positive relationship. Meanwhile, the correlation between knowledge and practices was relatively weak but statistically significant, with a ρ value of 0.085 (*p* = 0.006). Finally, attitudes showed a significant moderate positive correlation with practices, displaying a ρ value of 0.170 (*p* = 0.001). The correlation between attitudes and knowledge was also significant and positive.

**Table 8:** Correlation coefficients between knowledge, attitudes, and practices with respect to the dietary and lifestyle-related risk factors for colorectal cancer.

**Variables**	**Overall knowledge**		**Overall attitudes**		**Overall practices**	
	ρ	***p***	ρ	***p***	ρ	***p***
Overall knowledge	1	1	0.293	0.001^*^	0.085	0.006^*^
Overall attitudes	0.293	0.001^*^	1	1	0.170	0.001^*^
Overall practices	0.085	0.006^*^	0.170	0.001^*^	1	1

^*^Significant p-value.

## 4 Discussion

This study was conducted to investigate the KAP levels toward dietary and lifestyle-related risk factors for CRC and examine possible associations between the studied variables among the Saudi population. Despite an apparent increase in early-onset CRC with the majority of patients being diagnosed at an advanced stage (7), no previous KAP assessment of dietary and lifestyle-related risk factors for CRC has been reported. Therefore, the results of this study are anticipated to highlight areas of particular deficit in the current KAP levels, thus supplying healthcare providers with useful data to support the implementation of appropriate public health strategies and community programs to further improve the KAP levels, promote suitable modifications in dietary habits and lifestyle choices, and ultimately reduce the risk of CRC and enhance overall public health.

The study population included 1,040 participants with a notably diverse distribution across age groups (18–24 years: 29.0%; 25–39 years: 35.4%; 40–59 years: 30.2%). However, only 5.4% of the study's participants were aged 60 years or older. The sex distribution of the participants was also relatively well balanced (male: 45.1%; female: 54.9%). In addition, the population appeared generally healthy, with slightly more than one-third (35.4%) having normal BMI values, 79.5% being non-smokers, 78.2% reporting having no history of chronic diseases, 40.2% being fairly inactive, 84.3% reporting no food allergies, and few reporting having personal or family experience with CRC or polyps.

Despite the healthy profile of the study participants, the KAP assessment suggested that a majority of participants displayed low knowledge (77.8%), negative attitudes (78.6%), and poor practices (75.0%) with respect to the dietary and lifestyle-related risk factors for CRC. This raises concerns regarding the knowledge, attitudes, and practices among non-healthy individuals, whose scores are expected to be even lower. In addition, low KAP levels may contribute to the increasing incidence of early-onset CRC, as younger individuals with low KAP may lack awareness of risk factors such as poor diets, lack of exercise, and smoking, leading to the neglect of preventive measures. This finding highlights an issue that should be targeted via community and public health strategies aimed at improving knowledge, attitudes, and practices toward these risk factors for CRC to realize better health outcomes. Furthermore, the findings of this study are inconsistent with other recent studies conducted in Jordan (20) and the UAE (21), which reported high levels of knowledge. This may be attributable to different public health programs between countries. In addition, these studies were performed only among students, who typically have greater access to health education in schools/universities and through online sources, alongside more opportunities to participate in health awareness programs.

The 14 knowledge questions revealed varying levels of knowledge among the participants, with the “high knowledge” percentages ranging between 15.8% and 66.2% and the “low knowledge” percentages ranging between 33.8% and 84.2%. More than half of the participants displayed high knowledge in regard to several risk factors believed to promote the development of CRC, including the consumption of processed meat once per day or more (66.2%), smoking (65.6%), and being overweight or obese (62.9%).

High knowledge was found to be more common among female, single, had studied in a medical field, reported using media (TV and radio) or organizations as their primary source of dietary information, and having first/second-degree relatives diagnosed with CRC or CRC. This indicates that younger individuals may be more interested in health information, possibly owing to greater engagement with media and health/lifestyle campaigns (33). In addition, higher knowledge levels among females and participants from medical backgrounds have been reported previously in other studies (34, 35). The former finding may be the result of women tending to have higher medical service utilization than men (36, 37). Therefore, multilayered programs taking into account diverse demographic characteristics and personal experiences should be examined to develop effective public health strategies, especially considering that it has been reported that individuals who live in Saudi Arabia consume more meat (38), have a higher proportion of smokers (39), and have a high prevalence of obesity (40).

Conversely, more than half of the participants displayed low knowledge about the daily consumption of aspirin (84.2%) and supplements (vitamins, minerals, and herbs; 68.1%). The participants of this study have incorrect information and reported that it is recommended to take aspirin and supplements every day to reduce the CRC risk. This may be attributable to participants seeking health-related information from various sources and also being influenced by them (41). Thus, there exists a need to raise awareness about local and international guidelines pertaining to CRC, such as through the professional social media platforms of the Ministry of Health, as a means to provide better access to accurate and up-to-date information. In addition, the participants displayed insufficient knowledge about the risks associated with the low consumption of fruits and vegetables (fewer than five servings per day; 66.5%) and the excessive consumption of red meat (once per day or more; 63.8%). This indicates that the study participants may not have recognized the importance of consuming five portions per day of fruits and vegetables to provide essential nutrients such as vitamins, minerals, and fiber, which exert antioxidant, anti-inflammatory, and anticancer effects (42, 43). A recent study by Alnasser reported that most participants showed low awareness of the Healthy Food Palm and the Saudi Healthy Plate guidelines, with only 11.1% and 30.3%, respectively, able to identify guideline-associated visual illustrations (44). This suggests the need to increase awareness about these initiatives as a means to improve public health and mitigate the development of diseases such as CRC. Finally, the study participants displayed low knowledge of the differences in gender susceptibility to CRC between men and women (64.0%), which may be ascribed to challenges facing public health awareness and screening programs in Saudi Arabia (45).

Low knowledge was more common among participants who were 40–59 years old, male, had no specific study field, and reported using family members as their source of dietary information. These findings suggest an urgent need for public discussions and educational initiatives aimed at increasing people's knowledge about the dietary and lifestyle-related risk factors for CRC to foster healthier dietary and lifestyle choices to reduce disease risk.

The 12 attitude statements indicated differences in attitudes among participants, with the “positive attitude” percentages ranging between 56.6% and 88.5% and the “negative attitude” percentages ranging between 11.5% and 43.4%. The percentages of positive attitudes were more than 50% for all 12 statements; however, three of these statements were considered as representing negative attitudes toward CRC risk factors. These statements were “no, I don't need to modify my diet as I'm not at risk for CRC”, “I don't want to follow a healthy diet as it is not worthwhile for preventing CRC”, and “I should increase my intake of high-fat foods”, which may be related to the majority of the study population regarding themselves as healthy and not seeing themselves sufficiently at risk to need to modify their diet. Positive attitudes were more common among participants who were 18–24 or 25–39 years old, very active, reported using media (TV and radio) or organizations as their primary source of dietary information, and having personal or family experience with CRC or polyps. Similar findings have been reported previously (46–48).

Among the 31 food items, variations in dietary practices were observed, with the “good practice” percentages ranging between 20.2% and 72.9% and the “poor practice” percentages ranging between 27.1% and 79.8%. More than half of the participants showed good practices for 19 food items, including fresh fruit, canned fruit, juices with added sugar, fresh vegetables, leafy greens, cooked vegetables, legumes, whole grains, processed meats, white meat, non-meat protein, fast food, low-fat dairy products, carbonated beverages, carbonated beverages without sugar, sweets, canned/tinned foods, animal fats, and vegetable oils. Conversely, more than half of the participants showed poor practices for dried fruit, fresh fruit juice, salted nuts, starches, breakfast cereals, red meat, processed white meat, fish/seafood, tuna, full-fat dairy products, milk alternatives, and seeds. Having good practices was more common among participants who were 40–59 or ≥60 years old, married, employed, ex-smokers, and very active. This may be attributable to these groups having had more experiences and greater exposure to health awareness campaigns, coupled with higher motivation to improve their diets and lifestyles (48–51). Therefore, as diet, smoking, and physical activity are all modifiable lifestyle factors which can influence both CRC incidence and survival, the development of awareness and educational programs may enhance KAP levels regarding dietary habits and lifestyle factors linked to CRC risk (52, 53). These programs could, for example, highlight the importance of increasing dietary fiber and reducing meat and sugar, as well as the health benefit of maintaining a normal weight and being active and non-smoker. It has been reported that high fiber consumption from vegetables, fruits, whole grains, and cereals may decrease bowel transit time, thus decreasing the contact between carcinogens and the colonic epithelium. It also increases water content in feces, diluting potential carcinogens and promoting beneficial gut microbiota, which ferment fiber to produce short-chain fatty acids that exhibit tumor-suppressive effects (54). Furthermore, fruits and vegetables provide essential vitamins, minerals, folate, plant sterols, and protease inhibitors that have antioxidant and anti-inflammatory properties. These components can help prevent DNA and cellular damage, further reducing CRC risk (54).

On the other hand, high consumption of meat and sugar were associated with increased risk of CRC. High meat intake increase the occurrence of various carcinogenic compounds, including haem iron from red meat, exogenous N-nitroso compounds from processed meat, ionized fatty acids and secondary bile acids related to fat content in meats, as well as polycyclic aromatic hydrocarbons and heterocyclic amines formed during the high-temperatures cooking (55). In addition, excessive sugar intake can increase CRC risk, as glucose intake leads to a direct and pronounced insulin response, stimulating the release of insulin-like growth factor-I, which promotes cell growth and inhibits apoptosis, thereby enhancing CRC risk (56). Furthermore, physical activity is associated with a decreased risk of CRC by reducing whole-body and visceral fat, metabolic dysregulation, chronic inflammation, oxidative stress, and the enhanced immune (53). Moreover, smoking is also linked to an increased risk of CRC due to its nicotine content. Nicotine has been reported to increase cellular proliferation by altering receptor expression and phosphorylation patterns. The exposure to nicotine promotes the growth of CRC cells by upregulating acetylcholine and noradrenaline receptors (57).

The moderate positive relationship observed between knowledge and attitudes suggests that higher levels of knowledge are associated with more positive attitudes toward CRC risk factors. However, the relatively weak correlation observed between knowledge and practices indicates a small positive association, where higher knowledge is only slightly related to better practices. In addition, the moderate positive correlation between attitudes and practices indicates that more positive attitudes are associated with better practices related to CRC prevention. The significant and positive correlation between attitudes and knowledge reinforces that those with more positive attitudes tend to have higher knowledge levels.

To the best of our knowledge, the present work represents the first KAP assessment of the dietary and lifestyle-related risk factors for CRC in Saudi Arabia. In addition, as the sample size used in this study was sufficiently large, the obtained findings should be generalizable and suitable for utilization as a baseline for future research. However, several limitations mean that the study findings should be interpreted with caution. First, the cross-sectional study design only reveals the associations between variables but cannot prove causality. Second, even though convenience sampling is the most cost-effective method for collecting data from a large demographic group, the sociodemographic data of this study may not reflect the actual structure of the entire Saudi population. Third, the use of social media platforms such as WhatsApp and X to disseminate the online questionnaire may have introduced minor bias, resulting in underrepresentation of certain groups, particularly elderly individuals who may be less familiar with or have limited access to these platforms.

## 5 Conclusion

The present study offers valuable insights into the current KAP levels toward dietary and lifestyle-related risk factors for CRC and possible associations between the studied variables among the Saudi population. The obtained findings indicate insufficient KAP levels toward these risk factors for CRC. Thus, it is necessary to implement strategies to develop a better understanding of these risk factors among the Saudi population to reduce the public health and economic burden associated with CRC, especially in younger age groups. The results of this study could be used as a foundation for investigators, policymakers, and healthcare leaders to support future research and strategies to improve overall colon health. Further studies are warranted to assess KAP levels across different sociodemographic groups, particularly among younger populations. Such research would provide healthcare providers with the necessary information to implement effective educational intervention programs targeting various demographics, aiming to change behaviors and promote dietary habits and lifestyle modifications to reduce the risk of CRC.

## Data Availability

The original contributions presented in the study are included in the article; further inquiries can be directed to the corresponding authors.
